# Case Report of Foreign Body Stuck in Esophagus with Failure of Endoscopic Management in a Man with a History of Pica

**DOI:** 10.1155/2017/1934787

**Published:** 2017-11-01

**Authors:** Holly Mulinder, Allison Ammann, Yana Puckett, Sharmila Dissanaike

**Affiliations:** Department of General Surgery, School of Medicine, Texas Tech University, Lubbock, TX 79409, USA

## Abstract

This is a case report of foreign body ingestion in a 55-year-old intellectually disabled man with a history of pica and previous removal of ten plastic gloves from his rectum four months prior to this presentation. The patient presented after ingesting plastic gloves which formed large, rigid esophageal and gastric bezoars that were not amenable to endoscopic removal. An exploratory laparotomy and gastrostomy was performed, and a 10 × 4.5 × 2 cm gastric bezoar consisting of rigid plastic gloves was removed without complication. Special considerations must be taken when considering the ingestion of nonfood items in the intellectually disabled population as these cases may not present classically with symptoms of a gastric bezoar.

## 1. Introduction

Pica is the compulsive ingestion of food and nonfood items. It is a relatively common disorder in institutionalized patients with severe intellectual disability [1]. Prevalence of pica in this population has been reported as high as 25.8% [2]. Nonfood items ingested include dirt, paper, metal objects, and vinyl or latex gloves.

Complications of pica include malabsorption, constipation, vomiting, intestinal obstruction, lead and nicotine toxicity, parasitic infection, hepatitis, and requirement of surgical intervention [3]. Pica can result in the formation of a bezoar which untreated can result in bleeding, gastric ulcer due to pressure necrosis, perforation of intestine, and death.

Bezoars are most commonly formed in the stomach but may be formed in any part of the gastrointestinal tract. They are typically classified by their composition into four types: phytobezoars (vegetable matter), trichobezoars (hair), pharmacobezoars, and lactobezoars [4]. Bezoars composed of other materials such as vinyl or latex gloves, parasitic worms, and metals are less common.

Diagnosis of a bezoar can be made with computed tomography (CT) scans and esophagogastroduodenoscopy. Imaging can identify that a foreign body or bezoar is present but is unable to determine the material of the object. Gastric bezoars can often be diagnosed via endoscopy. A CT scan is most useful in patients presenting with small bowel bezoars as the site of obstruction within the small bowel can be visualized [4].

Chemical dissolution is an option for phytobezoars, but more invasive techniques are required for bezoars composed of other materials. While some gastric bezoars can be removed endoscopically, surgical removal with laparoscopy and gastrostomy may be required for definitive treatment of other cases [4]. Surgical removal is typically required for bezoars in the small bowel due to obstruction [5].

We present a case of a digestive tract bezoar composed of gloves that failed endoscopic treatment and required surgical management.

## 2. Case Presentation

The patient was a nonverbal 55-year-old male with a past medical history of severe intellectual disability, cerebral palsy, epilepsy, right eye impairment due to enucleation, severe chronic gastritis, grade B esophagitis, dysphagia, and pica behavior who presented with vinyl glove ingestion resulting in esophageal and gastric bezoars.

The previous year, the patient was placed on pica precautions with continued surveillance abdominal X-rays after three documented cases of pica activity in six months. The most recent pica-related event was four months prior to this admission when the patient presented with a rectal mass. At that time, ten plastic gloves were removed from the rectum via colonoscopy. Due to the patient's status, no verbal complaints were noted.

For this case, the patient presented to the gastroenterology clinic after an abdominal X-ray obtained for surveillance showed an esophageal bezoar of unknown etiology. At this time, no nausea, vomiting, constipation, or diarrhea was documented. After a period of observation, a flexible upper GI endoscopy was performed on hospital day 1 and showed a foreign body thought to be ingested vinyl gloves in the lower portion of the esophagus as well as in the gastric fundus. An endoscopic removal was attempted but due to the large size and stiffness of the bezoar, an endoscopic snare became entangled and the removal was abandoned. At this time, a decision was made to leave the patient's endotracheal tube in place due to the fear that removal could result in inhalation of the gloves into the patient's airway. The surgical team was consulted, and the patient was admitted to the surgical intensive care unit (SICU).

**Figure 1:**
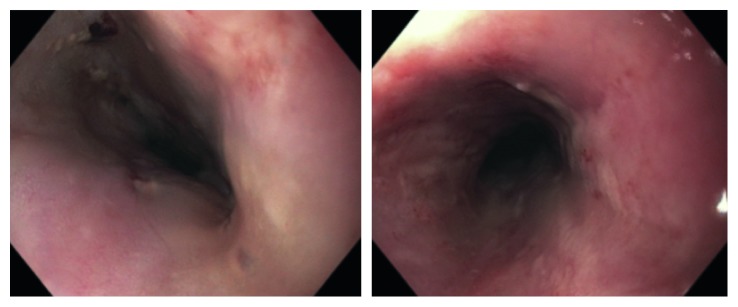
Images of esophagogastroduodenoscopy performed just prior to exploratory laparotomy and gastrostomy for evaluation of complete evacuation of foreign bodies in esophagus depicting irritation of the esophagus.

A direct laryngoscopy was then performed bedside with failure to visualize the bezoar. A CT chest/abdomen/pelvis was performed, and it showed a bezoar within the proximal esophagus at level of thyroid cartilage extending inferiorly to level of carina, measuring approximately 10 cm in craniocaudal length (Figure 1). It also showed an additional bezoar within the stomach measuring about 6 cm in transverse diameter and 6 cm in craniocaudal diameter.

**Figure 2:**
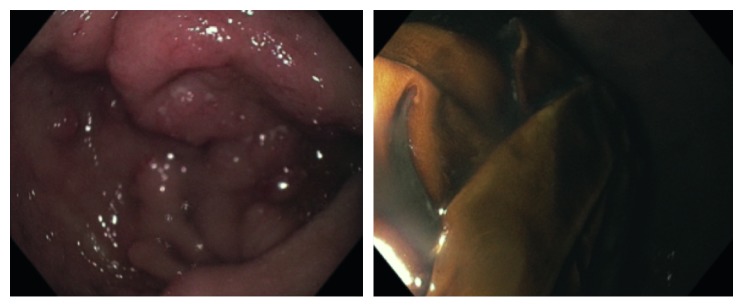
Esophagogastroduodenoscopy images of the antrum of the stomach depicting gastric hyperplasia and gastric bezoar composed of gloves.

**Figure 3:**
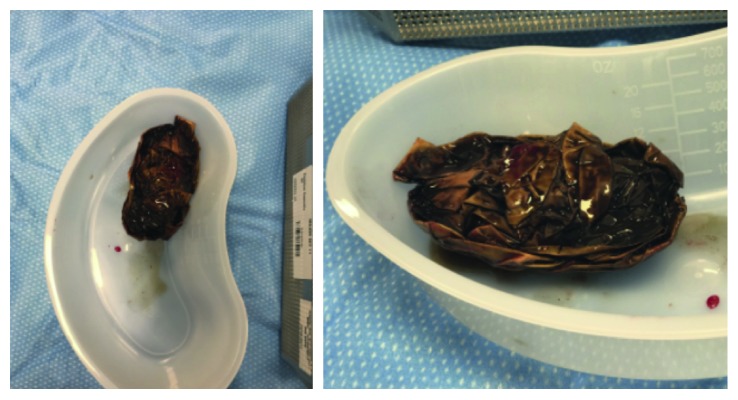
Images depicting the gastric bezoar composed of rigid plastic gloves that has just been removed via anterior gastrostomy.

Out of concern for esophageal damage, the esophageal bezoar was pushed into the stomach via rigid EGD. On hospital day 2, the patient underwent a repeat EGD (Figure 2) and exploratory laparotomy with gastrostomy and evacuation of gastric contents where a 10 × 4.5 × 2 cm bezoar of the plastic glove material was removed from the stomach (Figure 3). An open approach was used instead of a minimally invasive approach due to the nonpliable nature of the partially digested gloves, in order to extract the specimen it would require our incision to be approximately the same size as the open approach. The open approach also decreased contamination and infection risk because it allowed for the stomach contents to be removed outside the peritoneal cavity. The gastrostomy layer and fascia layer were closed, and the skin was left open with iodine-soaked kerlix. The patient was extubated in the operating room and transferred to the floor on postop day 1.

Postoperatively, the patient's course was uneventful. The patient received wound care in the hospital until the abdominal wound was closed on postop day 6. The following day, the patient was discharged to the state institution where he was previously residing.

## 3. Discussion

Bezoars composed of usual materials are seen more common in patients with severe intellectual disability than in the general population [1]. Diagnosing a bezoar in adults with severe intellectual disability is often challenging due to the lack of verbal complaints from the patients and unclear physical exam findings. Therefore, when these patients present with anorexia, constipation, vomiting, or change in behavior, it is important to have a high degree of suspicion for a bezoar. This is especially true in patients with a known history of pica. It is also important to note that some patients, such as the one presented in this case, may show no symptoms of a bezoar. Therefore, these patients with a known history of pica behavior should be considered for periodic surveillance with abdominal X-rays [3].

One retrospective study of five adult cases of ingested vinyl gloves found that when ingested, the gloves became stiff and nonpliable [5]. When multiple gloves were ingested together, the bezoar they formed was bulky and rigid resulting in increased risk of bleeding and perforation. A second study reviewing four cases of children ingesting vinyl gloves also reported hardening of the gloves upon ingestion [6].

Due to the unique composition of glove bezoars, treatment with endoscopic fragmentation and removal is not recommended. In these cases, there is an increased risk of snare entrapment and esophageal bleeding [5]. The optimal treatment for these patients is surgical laparotomy and gastrostomy. The postoperative complication rate of these procedures is low and is typically minor such as wound infection [7].

Prevention is an important component of the care of these patients. The institutions that care for these patients must strictly monitor the availability of materials such as gloves.

## 4. Conclusion

In conclusion, this case examined the ingestion of vinyl gloves in a 55-year-old severely intellectually disabled man with resultant formation of an esophageal and gastric bezoar. After endoscopic management failed, the bezoar was removed via exploratory laparotomy and gastrostomy. This case was unique because the patient had a history of intellectual disability with limited speech, pica, and previous removal of vinyl gloves from the rectum, and the bezoar removed was only discovered due to the implementation of surveillance abdominal X-rays. It is important to consider the material ingested when approaching removal of bezoars. In the case of vinyl glove ingestion, endoscopy has a limited role in the treatment of the resultant bezoar due to the rigid nature of the mass. A more invasive technique such as gastrostomy may be necessary if the mass created is too large to pass through the stomach or causes obstructive symptoms. Lastly, special considerations should be taken in the intellectually disabled population with a history of foreign body ingestion to monitor access to materials as well as symptoms of ingestion. In this population, some form of monitoring such as a KUB used in this case may be necessary to rule out foreign body ingestion when speech is limited.
